# Hepatitis C Virus NS2 Protein Inhibits DNA Damage Pathway by Sequestering p53 to the Cytoplasm

**DOI:** 10.1371/journal.pone.0062581

**Published:** 2013-04-30

**Authors:** Cintia Bittar, Shubham Shrivastava, Joydip Bhanja Chowdhury, Paula Rahal, Ratna B. Ray

**Affiliations:** 1 Department of Pathology, Saint Louis University, St. Louis, Missouri, United States of America; 2 Deaprtment of Biology, UNESP – São Paulo State University, São José do Rio Preto, São Paulo, Brazil; Rosalind Franklin University of Medicine and Science, United States of America

## Abstract

Chronic hepatitis C virus (HCV) infection is an important cause of morbidity and mortality globally, and often leads to end-stage liver disease. The DNA damage checkpoint pathway induces cell cycle arrest for repairing DNA in response to DNA damage. HCV infection has been involved in this pathway. In this study, we assess the effects of HCV NS2 on DNA damage checkpoint pathway. We have observed that HCV NS2 induces ataxia-telangiectasia mutated checkpoint pathway by inducing Chk2, however, fails to activate the subsequent downstream pathway. Further study suggested that p53 is retained in the cytoplasm of HCV NS2 expressing cells, and p21 expression is not enhanced. We further observed that HCV NS2 expressing cells induce cyclin E expression and promote cell growth. Together these results suggested that HCV NS2 inhibits DNA damage response by altering the localization of p53, and may play a role in the pathogenesis of HCV infection.

## Introduction

HCV is a hepatotropic virus belongs to family *Flaviviridae* and genus Hepacivirus. HCV genome contains a linear, positive-strand RNA molecule of ∼9,500 nucleotides [1]. A number of HCV genomes have been cloned, and sequence divergences indicate several genotypes and a series of subtypes for the virus [2]. In the United States, HCV genotypes 1a and 1b are predominant in patients with chronic infection [3]. An estimated 200 million people worldwide and 4 million people in the United States are infected with HCV. The development of end stage liver disease in individuals who are chronically infected with HCV is a growing problem worldwide. A number of HCV proteins have been implicated in the promotion of cell growth, both in vitro and in transgenic mouse models in the absence of inflammation or fibrosis [4]–[8], suggesting that persistent HCV infection and viral protein expression have a direct cancer-promoting effect.

The DNA damage checkpoint detects DNA damage and responds by activating signaling pathways, which cause a cell cycle halt while the damage is repaired or induce apoptosis if it cannot be repaired [9], [10]. When DNA damage is sensed, ataxia-telangiectasia mutated (ATM) phosphorylates and activates Chk2 at Thr-68 [11] that inhibits cell cycle progression. To maintain this cell cycle halt, p53 is phosphorylated and induces p21 [12]. Oncogene induced aberrant cell proliferation is also associated with DNA damage and checkpoint activation [13].

The mechanisms responsible for the progression of HCV infection to liver disease remain poorly defined. While indirect mechanisms, including chronic hepatic inflammation with associated oxidative stress and the potential for DNA damage, are likely to contribute to the development of hepatocellular carcinoma (HCC), strong evidence suggests that viral proteins are directly involved in HCV mediated pathogenesis [6], [8]. HCV infection has been shown to induce double stranded DNA breaks (DSBs) in hepatocytes [14]. In fact, several HCV proteins are involved in p53 and DNA damage sensor pathways. An association between HCV NS3/4A and ATM resulted in partial relocalization of ATM from nucleus to the perinuclear region [15], [16]. Overexpression of HCV NS3 may interact with p53 and modulates its downstream function [17]–[19]. HCV NS5A has been shown to associate with p53 and retains p53 in the cytoplasm [20], [21]. HCV NS2 is a non-structural transmembrane protein, anchored in the endoplasmic reticulum (ER) with a molecular weight of 23 kDa [22]–[24]. HCV NS2 along with NS3 constitutes a cysteine protease (NS2–3 protease) responsible for the cleavage between NS2 and NS3 which is required for HCV replication [25]–[27]. Although the essential role of HCV NS2 for the production of infectious virus is established [28], [29], additional functions of HCV NS2 protein are poorly understood. In this study, we investigated the role of HCV NS2 in DNA damage pathway. Our results suggested that the expression of HCV NS2 protein induces phospho-Chk2 and mislocalizes p53. HCV NS2 induces cyclin E expression and promotes cell proliferation. These findings suggest that NS2 may play an important role towards development of HCC.

## Materials and Methods

### Generation of Stable Cell Lines

HCV NS2 region was amplified by PCR and cloned into EcoRI and XbaI restriction sites of pcDNA3 mammalian expression vector. Primers used for PCR amplification are, sense: 5′ - CAG CGG GCG AAT TCA ATG GAC AC - 3′ and antisense: 5′ - ACG CCG TCT AGA CTA CAG CAA CCT C - 3′. HCV full length cDNA was cloned into XbaI and NotI restriction sites of mammalian expression vector. Recombinant DNA encoding HCV-full length, HCV NS5A or HCV-NS2 genomic region (HCV genotype 1a, H77 strain) was transfected into HepG2 cells. Vector DNA was used as a negative control. Transfected cells were selected with antibiotic G418 for three weeks, and colonies are pooled and used for subsequent study.

### Immunoblot Analysis

Protein lysates from HepG2 cells stably expressing HCV-FL, NS5A and NS2 were subjected to electrophoresis on polyacrylamide gel and were transferred onto a nitrocellulose membrane. The membrane was probed with an antibody specific for phospho-Chk2 (Thr-68), Chk2, γH2AX (Cell signaling), p53, p21 or cyclin E (Santa Cruz). Proteins were detected with an enhanced chemiluminescence western blot substrate (Pierce, Rockford, IL). The membrane was reprobed with actin for comparison of protein load in each lane.

### Immunofluorescence Assay

HepG2 stable cell line expressing HCV NS2 (HepG2-NS2) or full length genome (HepG2-FL) were washed in PBS, fixed with 3.7% formaldehyde for 20 min at room temperature, and blocked with 3% BSA for 1 hr. Fixed cells were permeabilized with 0.2% Triton X-100 for 5 min at room temperature, incubated with HCV NS2 specific mouse monoclonal antibody (generous gift by Charles Rice) overnight at 4°C. Cells were washed and incubated with anti-mouse Ig conjugated with Alexa 488 (Molecular Probes) for 1 h at room temperature. Finally, cells were washed and mounted for confocal microscopy (Olympus FV1000). For colocalization study, stable cell lines were stained with NS2 specific mouse monoclonal antibody and p53 specific rabbit polyclonal antibody followed by anti-mouse Ig conjugated with Alexa 488 and anti-rabbit Ig conjugated with Alexa 594 (Molecular Probes) respectively for immunofluorescence assay. HepG2 cells mock or transfected with plasmid DNA encoding HCV NS2 protein were stained with NS2 specific mouse monoclonal antibody and phospho-Chk2 (Thr 68) specific rabbit polyclonal antibody followed by anti-mouse Ig conjugated with Alexa 488 (Molecular Probes) and anti-rabbit Ig conjugated with Alexa 594 (Molecular Probes) secondary antibodies, respectively. Images were superimposed digitally for fine comparisons.

## Results and Discussion

### HCV NS2 Activates DNA Damage Checkpoint Pathway

**Figure 1:**
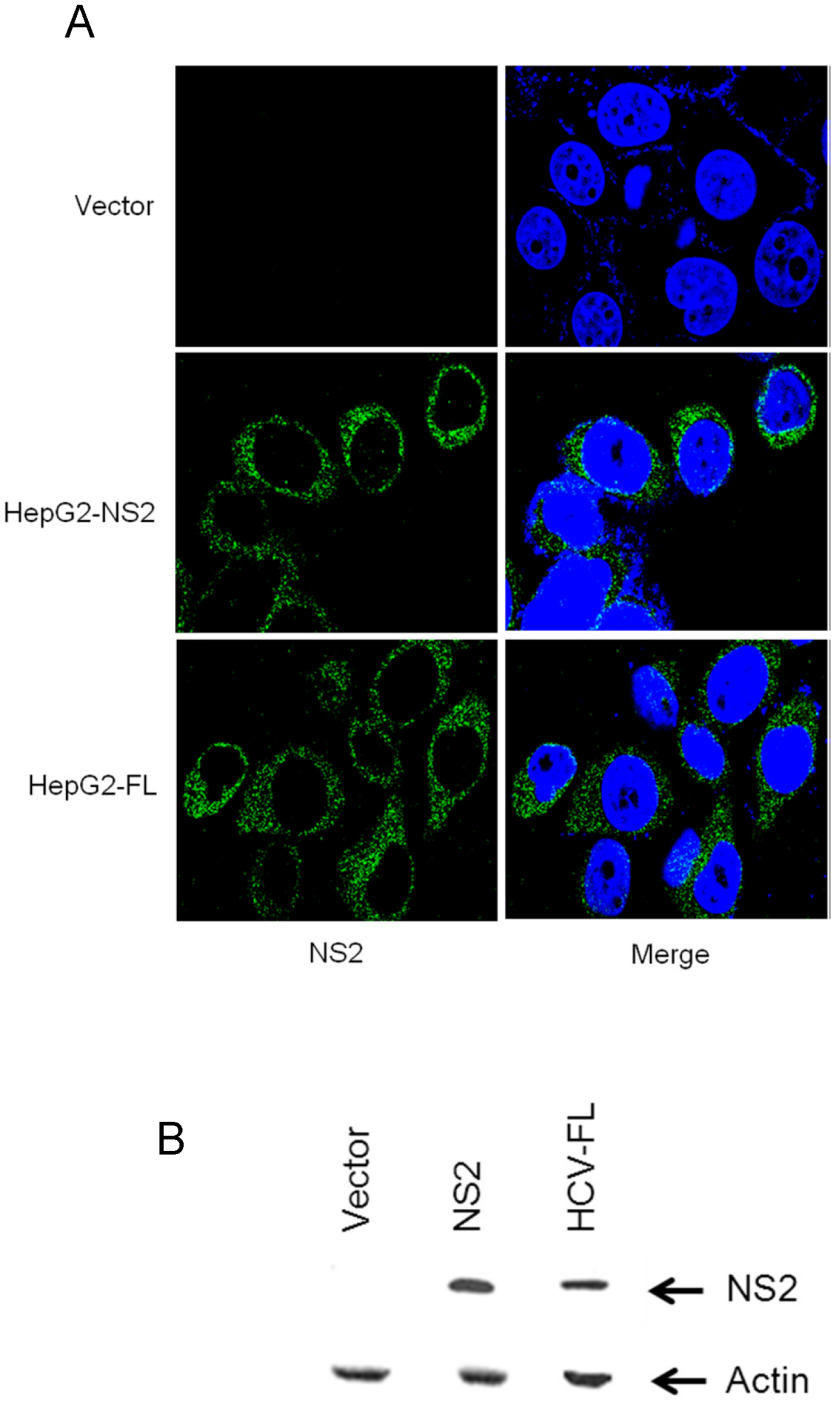
Generation of stable cell lines expressing HCV NS2 protein. (A) HepG2 cells are stably transfected with HCV-NS2 or HCV-FL. Immunofluorescence images displayed the expression of HCV NS2 (green color) in stably transfected HepG2 cells with HCV NS2 and HCV-FL. The nuclei (blue color) were stained with DAPI (4′, 6′-diamidino-2-phenylindole). Merged photographs are shown in right panel. (B) Cell lysates were prepared from HepG2 cells stably expressing HCV NS2 and HCV-FL. Protein expression of NS2 were analyzed by Western blot using specific antibodies. The blot was reprobed with actin for a comparison of protein load.

To study the role of HCV NS2 in DNA damage pathway, we have initially generated stable cell lines expressing HCV NS2 or HCV full-length (HCV-FL) cDNA in HepG2 cells. After selection with neomycin (G418), we collected the pooled cell lines for subsequent studies. Vector transfected pooled cells were used as control. Localization of HCV NS2 in stable transfectants was examined by immunofluorescence. Our results showed that HCV NS2 is expressed in cytoplasm (Figure 1A). We have also observed the expression of HCV NS2 in stable transfectants by western blot analysis (Figure 1B).

**Figure 2:**
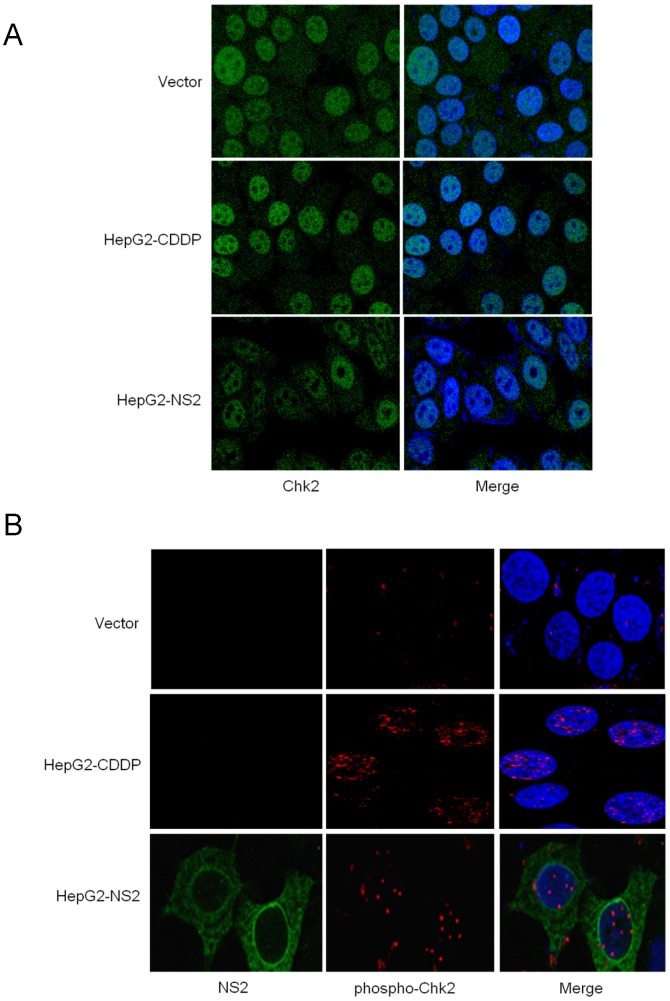
HCV NS2 induces nuclear foci formation of phosphorylated Chk2. (A) Confocal microscopy was performed for Chk2 localization in HepG2 cells stably expressing vector control or HCV NS2. HepG2 cells were treated with 15 µM cisplatin for 6 hrs and used as a positive control. Chk2 was stained with specific monoclonal antibody (green color) and nuclei were stained with DAPI (blue color). Merged photographs were shown in right panel. (B) Confocal microscopy was performed for phospho-Chk2 (Thr 68) localization in vector control and cisplatin treated HepG2 cells or HepG2 cells transfected with HCV NS2. Immunofluorescence images displayed the expression of HCV NS2 (green color) and phospho-Chk2 (red color) in control, cisplatin treated and HCV NS2 transfected HepG2 cells. The nuclei (blue color) were stained with DAPI. Merged photographs (right panel) were superimposed digitally for fine comparison.

**Figure 3:**
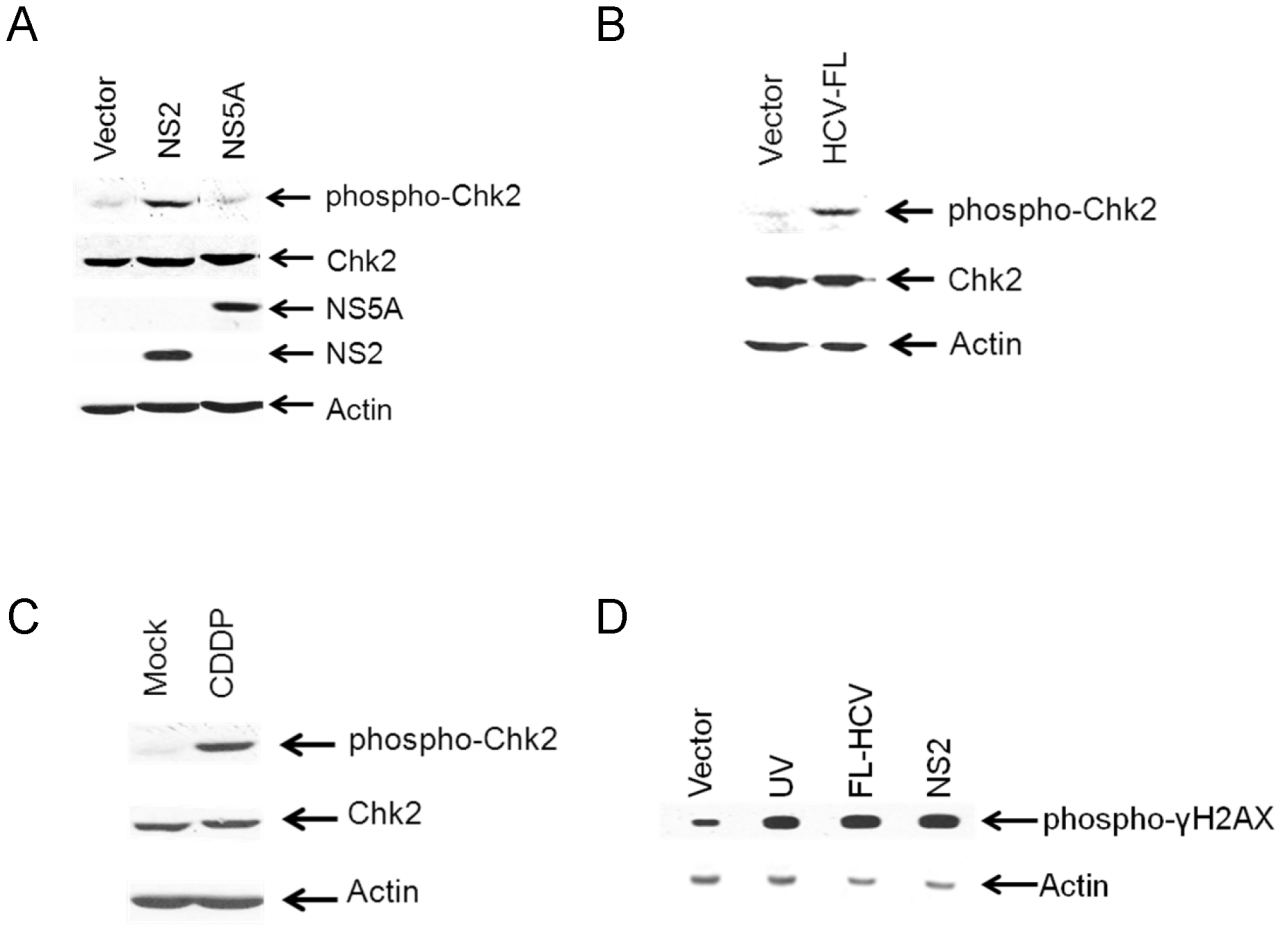
HCV NS2 activates Chk2 in hepatocytes. (A) Cell lysates were prepared from HepG2 cells stably expressing HCV NS2 and HCV NS5A. Protein expression of phospho-Chk2 (Thr-68), total Chk2, HCV proteins were analyzed by Western blot using specific antibodies. The blot was reprobed with actin for a comparison of protein load. (B) Cell lysates were prepared from HepG2 cells stably transfected with vector control or HCV-FL. Protein expression of phospho-Chk2, and total Chk2 were analyzed by Western blot using specific antibodies. The blot was reprobed with actin for a comparison of protein load. (C) Cell lysates were analyzed from mock or 15 µM cisplatin treated HepG2 cells, and protein expression of phospho-Chk2 and total Chk2 were analyzed by Western blot using specific antibodies. The blot was reprobed with actin for a comparison of protein load. (D) Cell lysates were prepared from HepG2 cells expressing HCV NS2 and HCV-FL. Protein expression of phospho-H2AX (Ser-139) was analyzed by Western blot using specific antibody. UV treated cell lysates were used as positive control. The blot was reprobed with actin for a comparison of protein load.

To examine the effect of DNA damage checkpoint pathway, we examined the subcellular localization of Chk2 and phospho-Chk2 (Thr 68) in control and HCV NS2 expressing cells. We have observed nuclear localization of Chk2 in control and HCV NS2 expressing cells (Figure 2A) as Chk2 is a nuclear protein kinase [30]. As expected, we observed nuclear localization of Chk2 protein in cisplatin treated HepG2 cells. Distinct nuclear foci of phospho-Chk2 were observed in HCV NS2 transfected HepG2 cells similar to cisplatin treated HepG2 cells whereas, very little phospho-Chk2 were observed in vector transfected HepG2 cells (Figure 2B). Our data showed that activated form of Chk2 localizes in distinct foci at the sites of DNA strand breaks in NS2 expressing cells. The stable HepG2 cell lines expressing HCV NS2, NS5A or HCV-FL were further examined for the phosphorylation of Chk2 by Western blot analysis using specific antibody. Cells expressing HCV NS2 displayed higher Chk2 phosphorylation when compared to the vector transfected control cells (Figure 3A). However, HCV NS5A did not showed any increase in Chk2 phosphorylation, suggesting that HCV NS2 protein has specific role in DNA damage pathway by upregulating Chk2 phosphorylation. Cells expressing HCV-FL also showed an increase in Chk2 phosphorylation (Figure 3B). HepG2 cells treated with cisplatin showed higher Chk2 phosphorylation and used as a positive control (Figure 3C). We did not observe modulation of total Chk2 protein expression. We further examined the DNA break by phosphorylation of H2AX by Western blot analysis. Our results suggested that HCV NS2 and HCV-FL expressing HepG2 cells display γH2AX at Ser^139^ when compared with vector expressing cells (Fig. 3D). We have used UV treated cells as a positive control for γH2AX expression.

### p53 Localizes in the Cytoplasm in the Presence of HCV NS2

**Figure 4:**
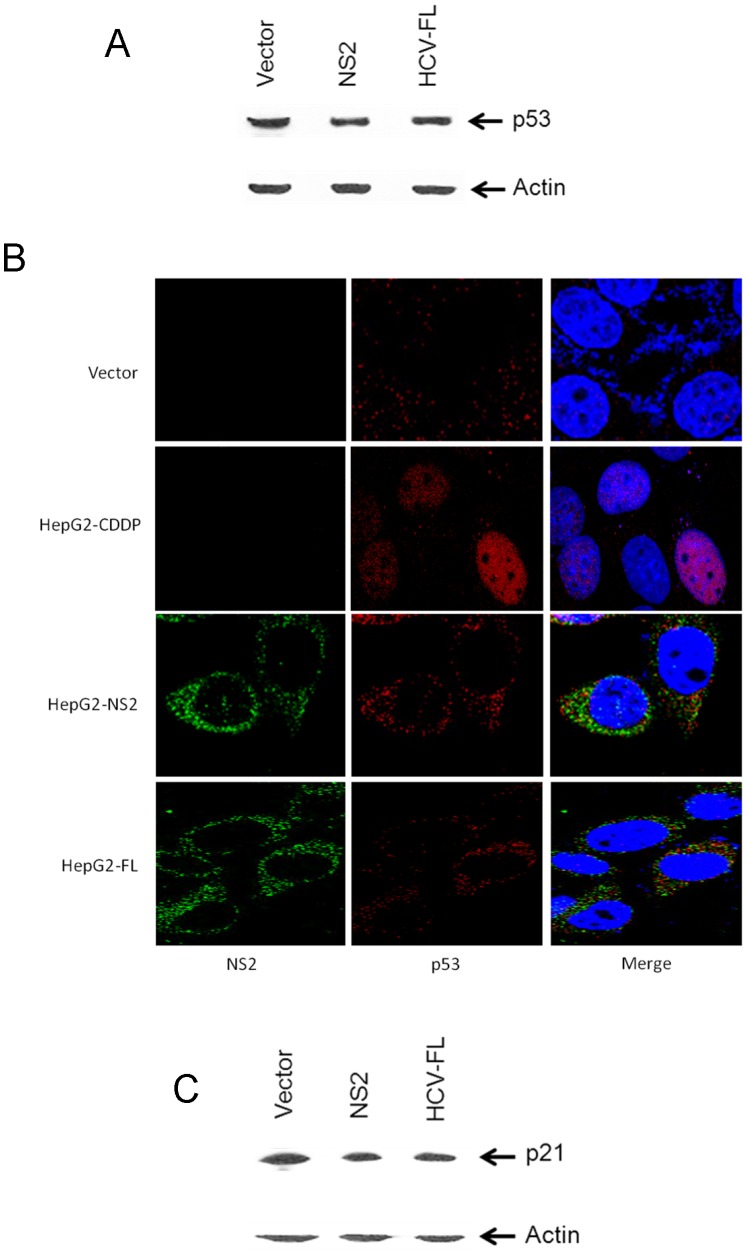
HCV NS2 does not activate p53 or its downstream molecule. (A) p53 expression was examined in HepG2 cells stably expressing HCV NS2 or HCV-FL and compared with control vector transfected cells using monoclonal antibody to p53. The blot was reprobed with an antibody to actin for comparison of equal protein load. (B) Immunofluorescence staining using a polyclonal antibody to p53 exhibits nuclear localization of p53 (red color) in mock-transfected cells (top row) or perinuclear localization of p53 in HCV NS2 (middle row) or HCV-FL (bottom row) transfected cells. HCV NS2 was stained with a monoclonal antibody (green color). The nuclei were stained with DAPI (blue color). Fluorescence images of left and middle panels were superimposed digitally for fine comparison (right panel). (C) p21 expression was examined in HepG2 cells stably expressing HCV NS2 or HCV-FL and compared with control vector transfected cells using monoclonal antibody to p21. The blot was reprobed with actin for comparison of equal protein load.

In response to DNA damage, Chk2 stabilizes the tumor suppressor protein, p53, by phosphorylating on Ser-20 that dissociates Mdm2 from p53 leading to cell cycle arrest in G1 phase [31]. Since DNA damage pathway involved p53, we chose HepG2 cells where wild type p53 is expressed. Huh-7 cells or its derivatives expressing HCV replicon or supporting HCV growth are widely used for a number of HCV related experiments, however, these cells express a high level of endogenous p53 with mutation at codon 220 (A:T–>G:C) [32]–[34]. Even treatment with DNA damaging agent did not show any changes in p53 expression level in Huh-7 cells [34]. Therefore, studying the functional consequences of p53-HCV protein association in Huh-7 cells may not generate the necessary information. Next we examined the status of phospho-p53 and total p53 in NS2 expressing HepG2 cells to determine the role of Chk2 in p53 stabilization. Interestingly, we did not observe phosphorylated p53 in HepG2 cells expressing HCV NS2 (data not shown). Subsequently, the blot was reprobed to examine the total p53 expression. Total p53 remained similar in control and HCV NS2 or HCV-FL expressing HepG2 cell lines (Figure 4A). Densitometry scanning suggested that there is no significant change in p53 level in HCV NS2 or HCV-FL expressing HepG2 cells as compared to vector control. Following activation of Chk2, it has been reported that p53 translocates into nucleus [35]. We further examined the p53 localization in HepG2 cells expressing HCV NS2 or HCV-FL by Immunofluorescence. Cells were fixed and stained with p53 rabbit polyclonal antibody and HCV NS2 mouse monoclonal antibody. Cytoplasmic localization of p53 was observed in HCV NS2 and HCV-FL expressing stable cell lines (Figure 4B), while control cells showed the nuclear localization of p53 as expected [36]. Although we observed that both HCV NS2 and p53 are localized in the cytoplasm, we did not observe a colocalization between these two proteins. It is possible that HCV NS2 retains p53 in the cytoplasm by associating with a cellular factor, which will be addressed in future study. As a positive control, we treated the HepG2 cells with CDDP and stained for p53 localization. Nuclear localization of p53 was observed. To block cell cycle progression, p53 also transcriptionally activates p21 [37]. To further confirm that p53 function is halted in HCV NS2 expressing cells, we examined the expression of p21, a downstream target of p53. There is no significant change of p21 protein expression in HepG2 cells stably expressing HCV NS2 or HCV-FL (Figure 4C).

### HCV NS2 Promotes Cell Proliferation

**Figure 5:**
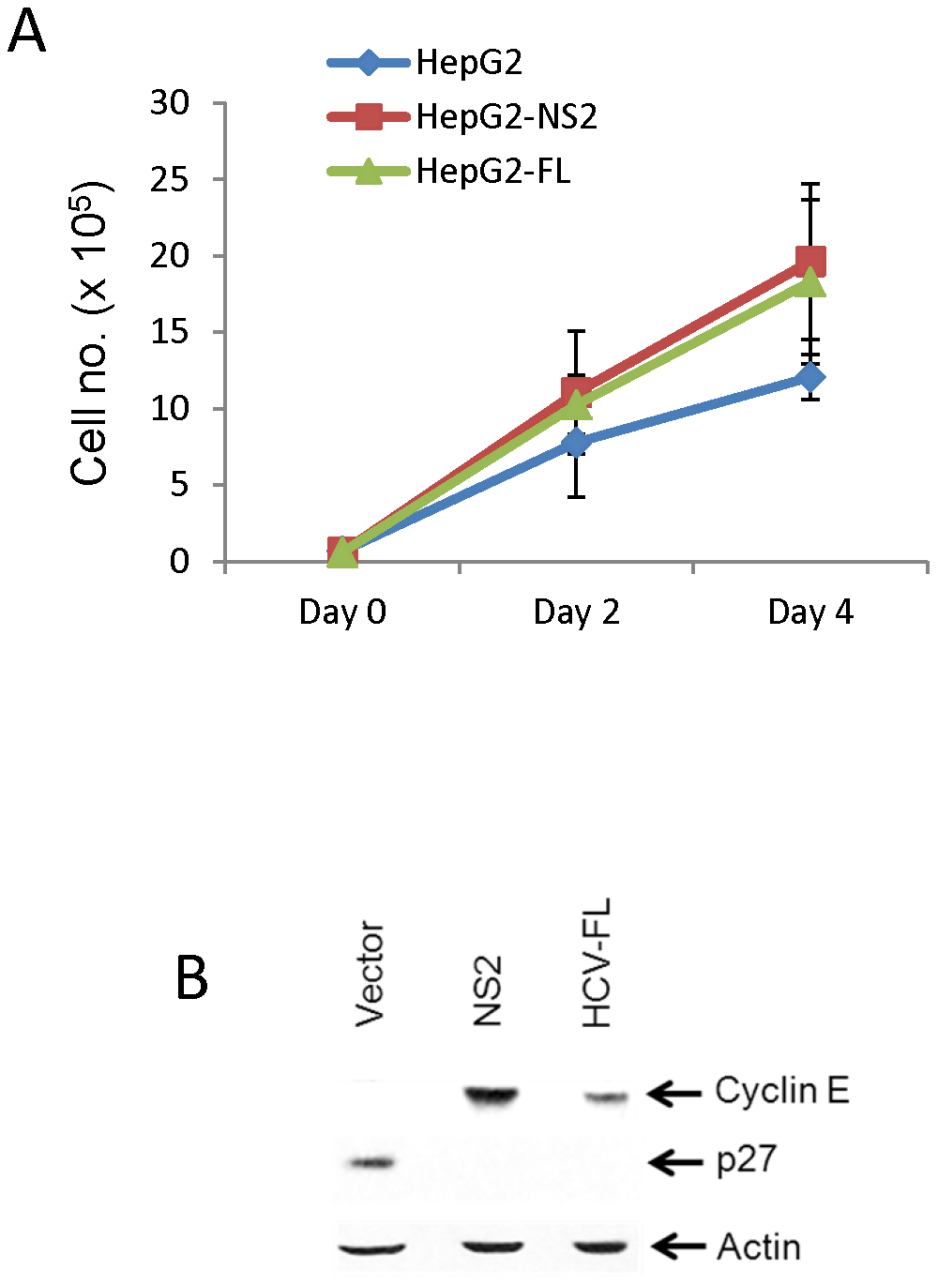
HCV NS2 promotes cellular proliferation. (A) Cell proliferation was measured in control, HCV NS2 and HCV-FL stably transfected HepG2 cells by trypan blue exclusion method. The number of viable cells was counted at day 2 and day 4. The results are presented as means of three different experiments with standard errors. (B) Cell lysates prepared from HCV NS2 or HCV-FL stably transfected HepG2 cells were analyzed by western blot for the expression of cyclin E and p27 with respect to control vector transfected cells using specific antibody. The blot was reprobed with actin for comparison of equal protein load.

Since HCV NS2 did not induce cell cycle arrest or apoptosis, we next examined the cell proliferation in HepG2 cells stably expressing HCV NS2 using trypan blue exclusion method. An increase in cell growth was observed in HepG2 cells expressing HCV NS2 or HCV-FL as compared to vector transfected control cells, suggesting that HCV NS2 enhances cell proliferation (Figure 5A). During cell proliferation, various cyclins associate with cyclin dependent protein kinases and regulate cell cycle progression through specific checkpoints. Cyclin E is known to interact with cdk2 and allow progression of cell cycle from G1 to S phase [38]. p27 is a cyclin dependent kinase inhibitor which inhibits Cdk4/cyclin D complex activity to cause G1 phase cell cycle arrest [39]. Cdk2/Cyclin E complex can phosphorylate p27 and lead to its degradation, allowing cells to transit from G1 to S phase [40]. It has been reported that aberrations in G1/S regulatory proteins are common in various tumors and aberrant expression of cyclin E and cyclin D1, downregulation of p16 and p27 as well as mutation of the retinoblastoma gene has frequently been observed in several cancers [41]. HCV core protein has also been reported to promote cell proliferation through upregulation of cyclin E expression levels [42]. Next, we examined the expression of cyclin E in HepG2 cells expressing HCV NS2 and HCV-FL. A significant increase of cyclin E was observed in presence of HCV NS2 as well as HCV-FL expressing cells (Figure 5B). As expected, we also observed downregulation of p27 in HepG2 cells expressing HCV NS2 or HCV-FL (Figure 5B). This result further verified that HCV NS2 protein expression may augment HepG2 cell proliferation.

In summary, HCV infection is associated with hepatocellular carcinoma, although the underlying mechanism is poorly understood. Hepatocyte transformation occurs by a combination of different factors such as environment, epigenetic, viral and genetic factors. In this study, we have shown that HCV NS2 expression induces DNA damage checkpoint branch by activating Chk2 phosphorylation. However, phosphorylation of Chk2 does not activate the downstream signaling molecules such as phosphorylation of p53 and activation of p21. Chk2 activation would normally lead to p53 stabilization and its accumulation in the nucleus. Interestingly, in HCV NS2 expressing cells, p53 retained in the cytoplasm that lead to blockage in DNA damage response pathway. Further, we have observed expression of γH2AX expression in HCV NS2 and HCV-FL expressing HepG2 cells. H2AX is one of key players in DNA damage repair (DDR) pathway, and its phosphorylation is considered as one of the signatures of DNA damage [43]. Cellular DDR machinery recognizes viral genome and is being utilized for viral replication for a number of viruses [43]. It is possible that HCV also utilized this DDR machinery for establishment of infection. Indeed the detail mechanism for triggering H2AX phosphorylation requires future investigation. We have also observed that HCV NS2 enhances cell proliferation and together these results suggest an important implication of HCV NS2 protein towards development of HCC. Thus it is possible that several HCV proteins are involved in HCV mediated pathogenesis by modulating p53 signaling pathways.
